# Gradual Elastic Suture (Shoelace Technique) Approximation and Platelet-Rich Plasma Infiltration Technique in the Closure of Open Fracture Wound and Infected Wound With Significant Skin Loss

**DOI:** 10.7759/cureus.30055

**Published:** 2022-10-08

**Authors:** Kashyap Kanani, Siddharth Jain, Aditya Pundkar, Rameez R Bukhari, Ankit Mittal

**Affiliations:** 1 Orthopaedics, Jawaharlal Nehru Medical College, Datta Meghe Institute Of Medical Sciences, Wardha, IND; 2 Orthopaedics, All India Institute of Medical Sciences, New Delhi, IND

**Keywords:** wound management, compound fractures, platelet rich plasma infiltration, infected wound, shoelace technique

## Abstract

Infected wounds can be really hard to manage in cases of open fractures, chronic osteomyelitis, and superficial infection. When the skin is damaged, bacteria can quickly enter the underlying tissue and cause a potentially fatal infection. Regular wound dressing with antimicrobial agents has become available in vials as a way to decrease the chance of bacterial colonization and infection and speed up the healing of wounds. In this report, we discuss the shoelace suture technique and platelet-rich plasma (PRP) infiltration in the wound. Due to its ability to stimulate and fasten the healing of wounds, PRP is becoming more and more popular. The progressive suture approximation (the shoelace technique) is an easy and effective technique for gently approximating the skin borders. The cytokines and growth factors in PRP play a critical role in the healing process. Hence, the combination of these two techniques will reduce the need for hospitalization, lead to better aesthetic outcomes, and reduce healthcare costs.

## Introduction

Infected and contaminated wounds with compound fractures are difficult to manage and may lead to various complications. It is very crucial to employ proper techniques and methods to manage these wounds [1]. Platelet-rich plasma (PRP) has the ability to stimulate and accelerate tissue regeneration. PRP is an autologous biological product made from a patient's blood in which a plasma fraction is obtained, and due to its ability to stimulate and fasten the healing of wounds, it is gaining more interest nowadays [2]. Cytokines and different growth factors like platelet-derived growth factor (PDGF), transforming growth factor β (TGF-β), vascular endothelial growth factor (VEGF), epidermal growth factor (EGF), insulin-like growth factor (IGF), and fibroblast growth factor (FGF) play important roles in the wound-healing process [3]. There are different modalities to manage these wounds, such as regular dressing with antibiotic ointments, collagen dressing, vacuum-assisted closure, local antibiotic infiltration, and many more [4]. In this report, we demonstrate the use of PRP infiltration along with the shoelace suture technique in the management of contaminated wounds and wounds of compound fractures [5]. The use of these techniques reduced the need for hospitalization, led to better aesthetic results, and reduced healthcare costs.

## Case presentation

**Figure 1:**
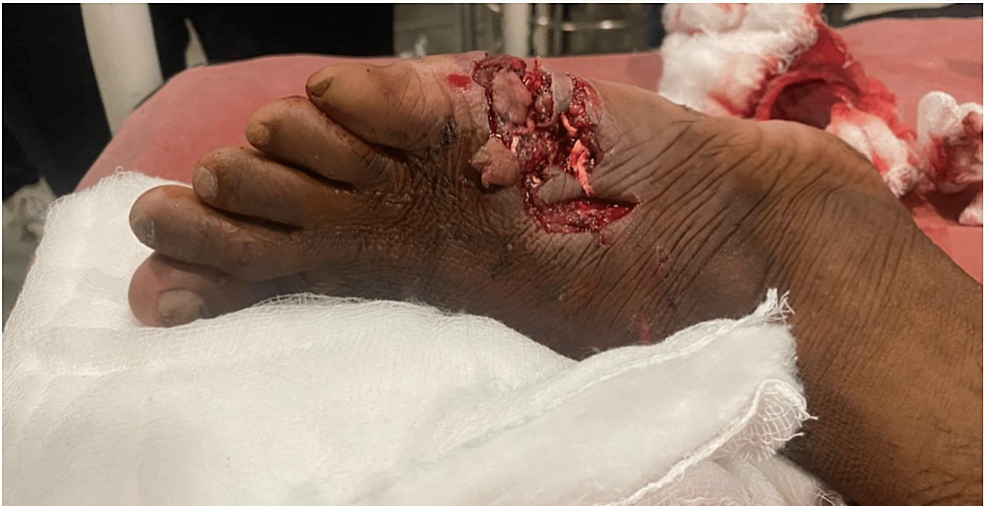
Initial presentation of the wound - superior view

**Figure 2:**
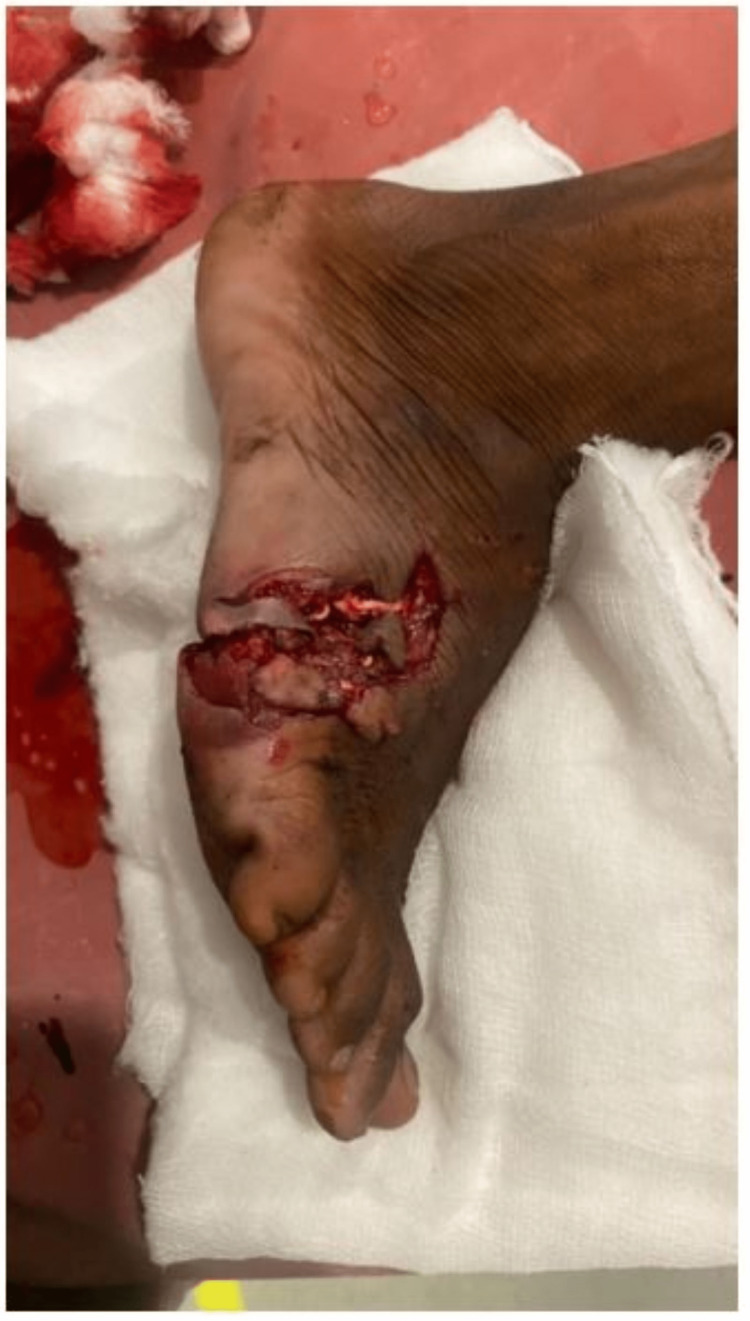
Initial presentation of the wound - lateral view

A 22-year-old male presented to the casualty with a history of trauma to his right foot due to falling off a metal block while working in a factory, following which he had developed contused lacerated wound over the anterolateral and posterolateral aspects of his right foot. On examination, an approximately 10 x 2 cm lacerated wound was present over the dorsolateral aspect of the right foot extending to the plantar aspect with severe contamination and skin loss (Figures 1, 2).

**Figure 3:**
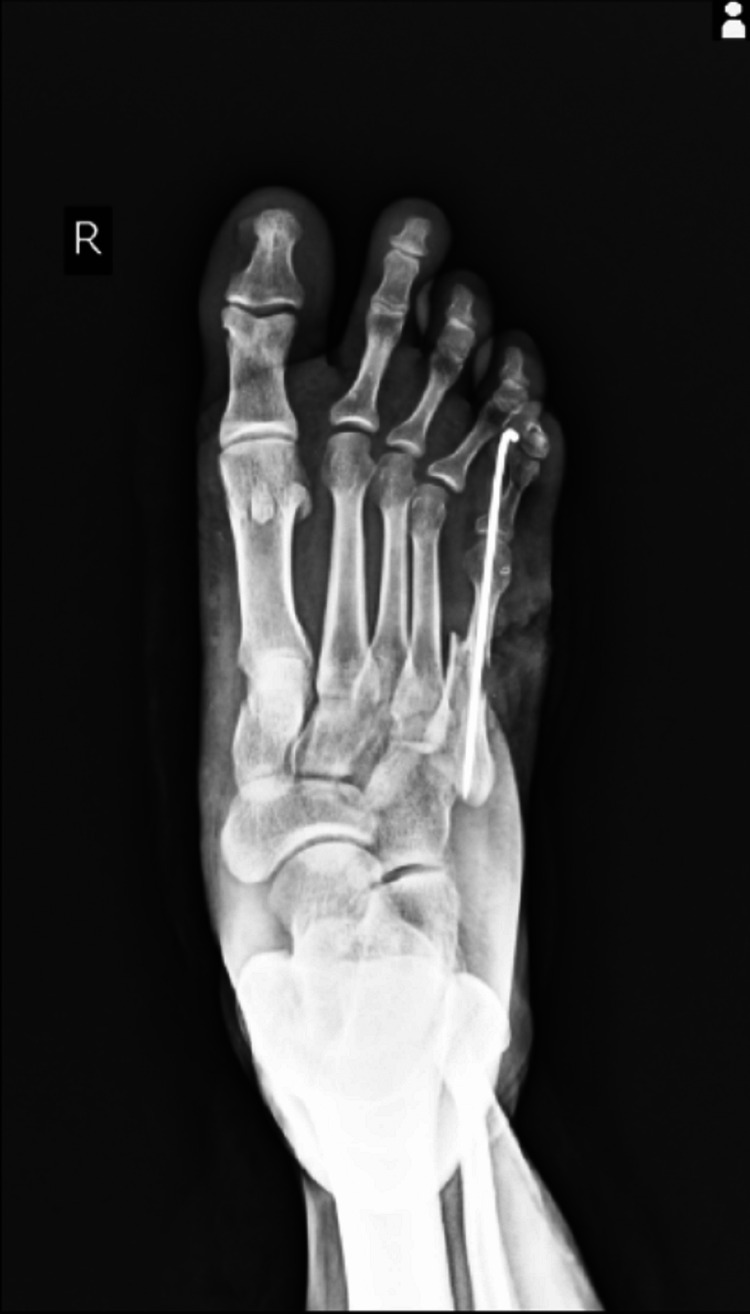
X-ray showing fifth metatarsal fracture managed with K-wire fixation - anteroposterior view

**Figure 4:**
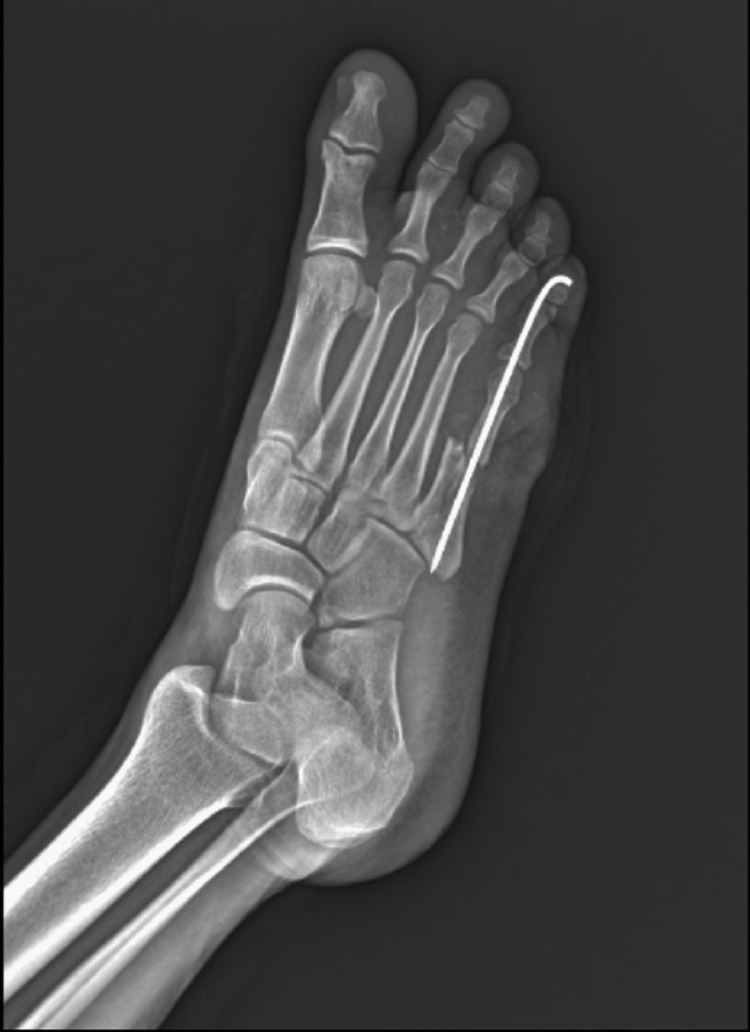
X-ray showing fifth metatarsal fracture managed with K-wire fixation - oblique view

**Figure 5:**
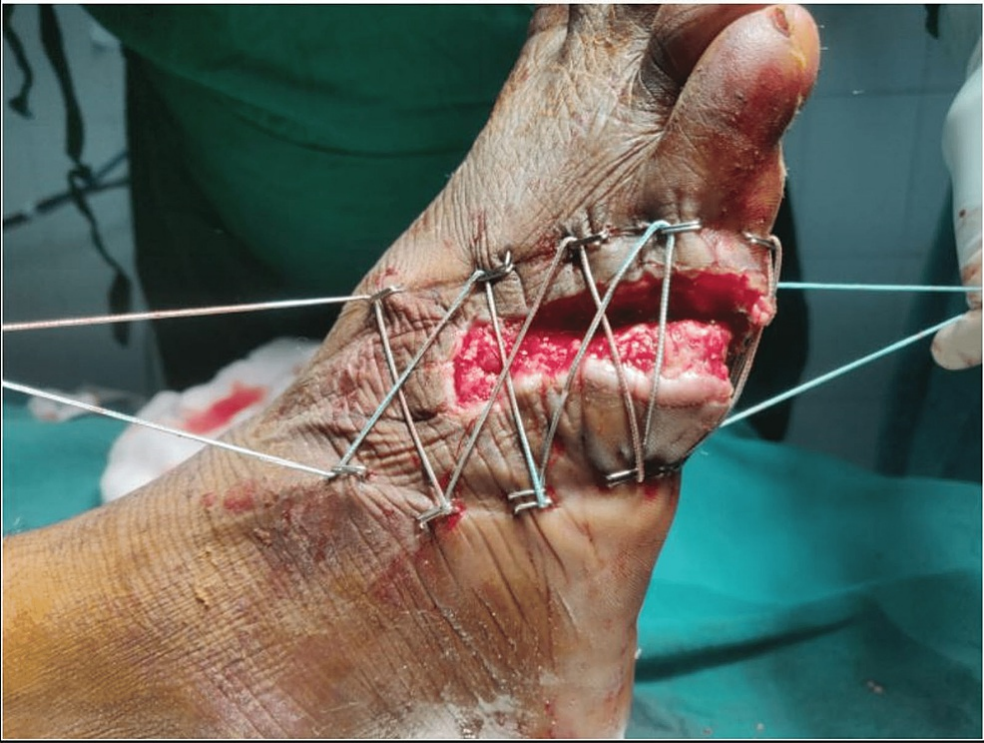
PRP infiltration and application of shoelace suture - lateral view PRP: platelet-rich plasma

**Figure 6:**
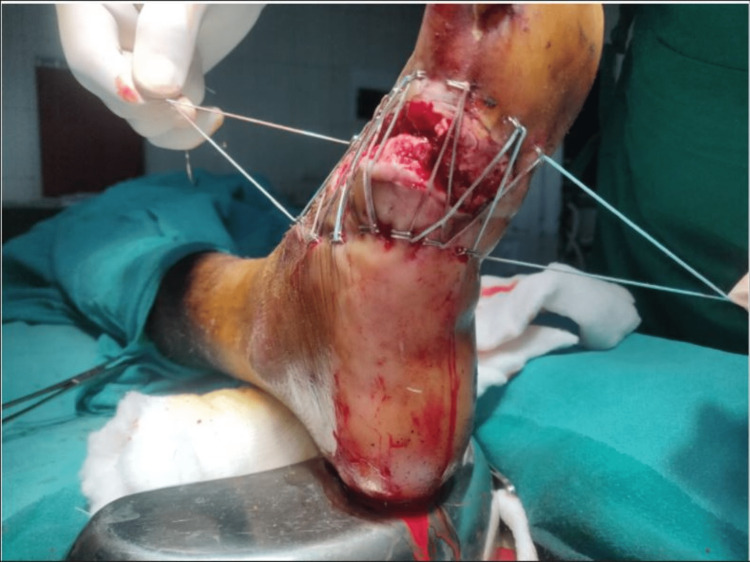
PRP infiltration and application of shoelace suture - plantar view PRP: platelet-rich plasma

**Figure 7:**
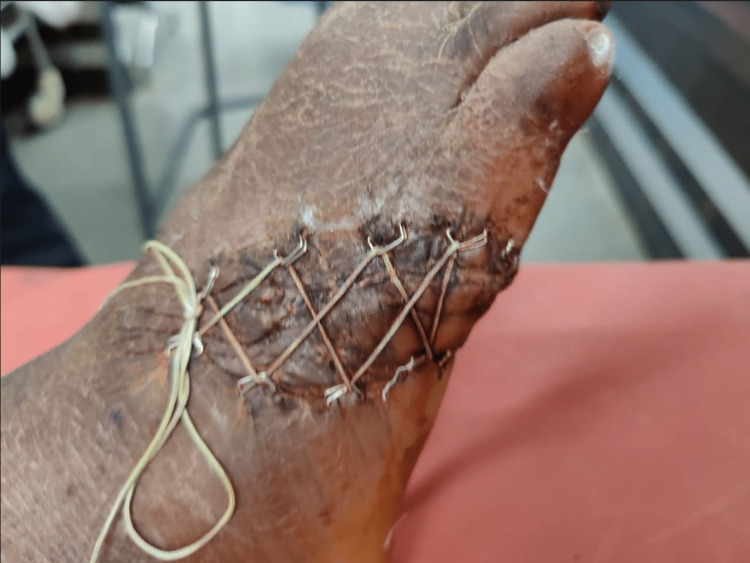
Final follow-up image of the wound after two weeks with tightening of shoelace every 48 hours and PRP infiltration every 96 hours - superior view PRP: platelet-rich plasma

**Figure 8:**
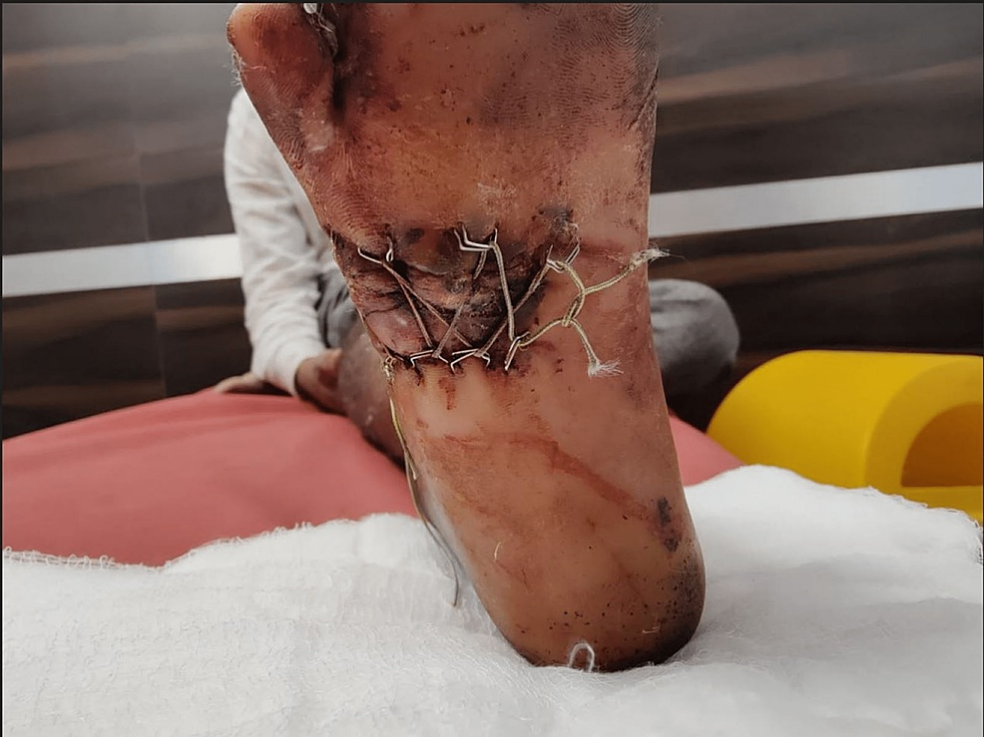
Final follow-up image of the wound after two weeks with tightening of shoelace every 48 hours and PRP infiltration every 96 hours - plantar view PRP: platelet-rich plasma

On palpation, there was tenderness, and abnormal mobility of the fifth metatarsal and crepitus was present. The patient was diagnosed with a Gustilo-Anderson type IIIB fracture of the fifth metatarsal right side. The patient was taken into the operation theater and was managed with debridement and shoelace suture application technique using Ethibond 5.0 and a skin stapler, and K-wire fixation was done for the fifth metatarsal fracture. The patient was given two doses of intravenous broad-spectrum antibiotics postoperatively. Whole blood obtained from the vein puncture was centrifuged at 1200 rpm for 10 minutes for the first spin and supernatant plasma containing platelets was transferred into another sterile tube (without anticoagulant). This supernatant plasma was centrifuged at 2000 rpm for 10 minutes for a second spin and leucocyte-rich PRP was collected. The patient was managed with PRP infiltration at the edges of the wound on the very next day. The patient was reviewed every 48 hours in the Orthopaedics Outpatient Department for further tightening of the shoelace suture. He was taken up for PRP infiltration every 96 hours in the Orthopaedics Outpatient Department. K-wire was removed and the patient was allowed full weight-bearing mobilization after six weeks. Figures 3-8 present images that encompass the span of the entire treatment course.

## Discussion

An infected wound is defined as a localized defect or excavation of the skin or underlying soft tissue when pathogenic organisms have invaded the live tissue surrounding the wound. When a wound becomes infected, the body's immune response is set off, resulting in inflammation and tissue damage as well as obstructing the healing process. Wound infection, if not treated properly, can increase medical costs and induce secondary complications. An incorrect repair process can result in serious damage, such as skin loss, infection, further impairment to adjacent tissues, and even systemic harm. Infection is the most prevalent and unavoidable hindrance to wound healing, especially in the case of chronic wounds and compound fractures. Antibiotic overuse can cause organ toxicity and systemic toxicity. A variety of cell types, growth factors, cytokines, and chemokines interact throughout the dynamic tissue repair process known as wound healing. If this mechanism is disturbed, it can lead to chronic inflammatory phase arrest, which can lead to persistent, non-healing wounds or excessive granulation tissue production.

All medical professionals involved in wound care and the management of complex fractures must be well-versed in the prevention and treatment of wound infection. There are various techniques of wound management, such as daily dressing, dressing with superficial antibiotic ointment application, intravenous antibiotics, local antibiotics, vacuum-assisted closure, and PRP infiltration. The use of the shoelace technique in the approximation of skin in fasciotomy wounds has been found effective [5]. PRP has the ability to stimulate and accelerate tissue regeneration. PRP is an autologous biological product made from a patient's blood in which a plasma fraction is obtained after centrifugation that has a higher platelet concentration than circulating blood [6]. With their hemostatic action, cytokines, and growth factors, platelets contribute significantly to the healing of wounds [7]. Thus, here we used the combination of the shoelace technique and PRP infiltration technique to promote secondary healing and for the closure of the open fracture wound without any long-term antibiotic administration. Several growth factors have been identified as being involved in the wound-healing process. The use of the shoelace suture technique along with PRP infiltration can be of great help in the healing process and approximation of the wound. We presented a case that was managed with PRP infiltration and the shoelace suture technique, which reduced the length of hospitalization, led to better aesthetic results, and reduced healthcare costs. In order to identify the precise clinical success of this technique and highlight patient benefits, additional research with larger patient cohorts and a control group is needed. Accident and trauma patients stand to gain the most from these techniques.

## Conclusions

In this case, a delayed closure was conceivable. The patient was regularly monitored every 48 hours for further tightening of the shoelace suture and the time from the first surgery to the closure was 14 days. The patient was regularly monitored in the fracture clinic for three weeks until sufficient soft tissue healing had occurred. There were no problems with superficial or deep infection while using this method. The patient was allowed weight-bearing mobilization after six weeks of the operative procedure. The patient did not undergo any other therapy and did not show any clinical signs of infection or nonunion.
